# The Changing Landscape of Neonatal Diabetes Mellitus in Italy Between 2003 and 2022

**DOI:** 10.1210/clinem/dgae095

**Published:** 2024-02-26

**Authors:** Novella Rapini, Maurizio Delvecchio, Mafalda Mucciolo, Rosario Ruta, Ivana Rabbone, Valentino Cherubini, Stefano Zucchini, Stefano Cianfarani, Elena Prandi, Riccardo Schiaffini, Carla Bizzarri, Barbara Piccini, Giulio Maltoni, Barbara Predieri, Nicola Minuto, Rossella Di Paola, Mara Giordano, Nadia Tinto, Valeria Grasso, Lucia Russo, Valentina Tiberi, Andrea Scaramuzza, Giulio Frontino, Maria Cristina Maggio, Gianluca Musolino, Elvira Piccinno, Davide Tinti, Paola Carrera, Enza Mozzillo, Marco Cappa, Dario Iafusco, Riccardo Bonfanti, Antonio Novelli, Fabrizio Barbetti, Diabetes Study Group of Italian Society for Pediatric Endocrinology and Diabetes (ISPED), Luciano Beccaria, Francesco Candia, Vittoria Cauvin, Roberta Cardani, Francesca Cardella, Anna Favia, Francesco Gallo, Patrizia Garzia, Paolo Ghirri, Stefania Innaurato, Lorenzo Iughetti, Nicola Laforgia, Donatella Lo Presti, Alberto Marsciani, Franco Meschi, Rossana Panzeca, Bruno Pasquino, Roberta Pesavento, Giulia Pezzino, Petra Reinstadler, Carlo Ripoli, Silvia Savastio, Tiziana Timpanaro, Stefano Tumini, Gianni Vento

**Affiliations:** Monogenic Diabetes Clinic, Endocrinology and Diabetes Unit, Bambino Gesù Children's Hospital, IRCCS, 00165 Rome, Italy; Metabolic Disorder and Diabetes Unit, “Giovanni XXIII” Children Hospital, 70100 Bari, Italy; Unit of Pediatrics, Department of Biotechnological and Applied Clinical Sciences, University of L'Aquila, 67100 L'Aquila, Italy; Translational Cytogenomics Research Unit, Laboratory of Medical Genetics, Bambino Gesù Children's Hospital, IRCCS, 00146 Rome, Italy; Translational Cytogenomics Research Unit, Laboratory of Medical Genetics, Bambino Gesù Children's Hospital, IRCCS, 00146 Rome, Italy; Department of Health Sciences, Division of Pediatrics, University of Eastern Piedmont, 28100 Novara, Italy; Pediatric Endocrinology and Diabetology Unit, Department of Women's and Children's Health, Azienda Ospedaliero Universitaria delle Marche, G. Salesi Hospital, 60126 Ancona, Italy; Pediatric Endocrine Unit, University Hospital of Bologna Sant’Orsola-Malpighi, 40138 Bologna, Italy; Endocrinology and Diabetes Unit, Bambino Gesù Children's Hospital, IRCCS, 00165 Rome, Italy; Department of Systems Medicine, University of Rome Tor Vergata, 00133 Rome, Italy; Department of Women's and Children's Health, Karolinska Institutet, 17177 Stockholm, Sweden; Pediatrics Clinic, University of Brescia and ASST Spedali Civili of Brescia, 25123 Brescia, Italy; Endocrinology and Diabetes Unit, Bambino Gesù Children's Hospital, IRCCS, 00165 Rome, Italy; Endocrinology and Diabetes Unit, Bambino Gesù Children's Hospital, IRCCS, 00165 Rome, Italy; Endocrinology and Diabetology Unit, Meyer University Children's Hospital IRCCS, 50139 Florence, Italy; Pediatric Endocrine Unit, University Hospital of Bologna Sant’Orsola-Malpighi, 40138 Bologna, Italy; Department of Medical and Surgical Sciences of Mother, Children and Adults, Pediatric Unit, University of Modena and Reggio Emilia, 41124 Modena, Italy; Regional Center for Pediatric Diabetes, IRCCS Istituto Giannina Gaslini, 16147 Genoa, Italy; Research Unit of Diabetes and Endocrine Diseases, Fondazione IRCCS Casa Sollievo della Sofferenza, 71013 San Giovanni Rotondo, Italy; Department of Health Sciences, University of Eastern Piedmont, 28100 Novara, Italy; Laboratory of Genetics, “Maggiore della Carità” Hospital, 28100 Novara, Italy; Department of Molecular Medicine and Medical Biotechnology, University of Naples Federico II/CEINGE Advanced Biotechnologies Franco Salvatore, 80131 Naples, Italy; Department of Experimental Medicine, University of Rome Tor Vergata, 00133 Rome, Italy; Department of Experimental Medicine, University of Rome Tor Vergata, 00133 Rome, Italy; Pediatric Endocrinology and Diabetology Unit, Department of Women's and Children's Health, Azienda Ospedaliero Universitaria delle Marche, G. Salesi Hospital, 60126 Ancona, Italy; Diabetes and Endocrine Service, Pediatric Unit, ASST Cremona, Maggiore Hospital, 26100 Cremona, Italy; Department of Pediatrics, Pediatric Diabetology Unit, Diabetes Research Institute, IRCCS Ospedale San Raffaele, 20132 Milan, Italy; Department PROMISE “G. D'Alessandro”, University of Palermo, 90127 Palermo, Italy; Growth Disorders, Endocrinology and Diabetology Clinic, Filippo del Ponte Pediatric Hospital, ASST Sette Laghi, 21100 Varese, Italy; Metabolic Disorder and Diabetes Unit, “Giovanni XXIII” Children Hospital, 70100 Bari, Italy; Department of Pediatrics, University of Turin, 10126 Turin, Italy; Genomics for the Diagnosis of Human Pathologies, San Raffaele Scientific Institute, Center for Omics sciences @OSR, 20132 Milan, Italy; Laboratory of Molecular Genetics and Cytogenetics, San Raffaele Scientific Institute, 20132 Milan, Italy; Department of Translational Medical Science, Section of Pediatrics, Università degli Studi di Napoli Federico II, 80131 Naples, Italy; Research Area for Innovative Therapies in Endocrinopathies, Bambino Gesù Children's Hospital, IRCCS, 00165 Rome, Italy; Department of Pediatrics, University of Campania Luigi Vanvitelli, 81100 Naples, Italy; Department of Pediatrics, Pediatric Diabetology Unit, Diabetes Research Institute, IRCCS Ospedale San Raffaele and Vita Salute San Raffaele University, 20132 Milan, Italy; Translational Cytogenomics Research Unit, Laboratory of Medical Genetics, Bambino Gesù Children's Hospital, IRCCS, 00146 Rome, Italy; Monogenic Diabetes Clinic, Endocrinology and Diabetes Unit, Bambino Gesù Children's Hospital, IRCCS, 00165 Rome, Italy

**Keywords:** monogenic diabetes, neonatal diabetes mellitus, congenital severe insulin resistance, autoimmune neonatal diabetes mellitus, molecular genetics

## Abstract

**Context:**

In the last decade the Sanger method of DNA sequencing has been replaced by next-generation sequencing (NGS). NGS is valuable in conditions characterized by high genetic heterogeneity such as neonatal diabetes mellitus (NDM).

**Objective:**

To compare results of genetic analysis of patients with NDM and congenital severe insulin resistance (c.SIR) identified in Italy in 2003-2012 (Sanger) vs 2013-2022 (NGS).

**Methods:**

We reviewed clinical and genetic records of 104 cases with diabetes onset before 6 months of age (NDM + c.SIR) of the Italian dataset.

**Results:**

Fifty-five patients (50 NDM + 5 c.SIR) were identified during 2003-2012 and 49 (46 NDM + 3 c.SIR) in 2013-2022. Twenty-year incidence was 1:103 340 (NDM) and 1:1 240 082 (c.SIR) live births. Frequent NDM/c.SIR genetic defects (*KCNJ11*, *INS*, *ABCC8*, 6q24, *INSR*) were detected in 41 and 34 probands during 2003-2012 and 2013-2022, respectively. We identified a pathogenic variant in rare genes in a single proband (*GATA4*) (1/42 or 2.4%) during 2003-2012 and in 8 infants (*RFX6*, *PDX1*, *GATA6*, *HNF1B*, *FOXP3*, *IL2RA*, *LRBA*, *BSCL2*) during 2013-2022 (8/42 or 19%, *P* = .034 vs 2003-2012). Notably, among rare genes 5 were recessive. Swift and accurate genetic diagnosis led to appropriate treatment: patients with autoimmune NDM (*FOXP3*, *IL2RA*, *LRBA*) were subjected to bone marrow transplant; patients with pancreas agenesis/hypoplasia (*RFX6*, *PDX1*) were supplemented with pancreatic enzymes, and the individual with lipodystrophy caused by *BSCL2* was started on metreleptin.

**Conclusion:**

NGS substantially improved diagnosis and precision therapy of monogenic forms of neonatal diabetes and c.SIR in Italy.

Neonatal diabetes mellitus (NDM) is a subtype of monogenic diabetes with onset within 6 months of age (1). NDM may be isolated or syndromic, depending on the single gene involved and its mode of inheritance can be autosomal dominant, autosomal recessive, or X-linked. In addition, most cases of NDM transient subtype (TNDM), which remits within weeks/months from diabetes outset, are associated with aberrations of chromosome 6q24 (1). NDM genes affect pancreatic beta cell development and/or function that cause severe insulin deficiency, with patients often times presenting with diabetic ketoacidosis (1). Individuals with congenital severe insulin resistance (c.SIR) show syndromic features such as lipodystrophy, acanthosis nigricans, and hirsutism and present with nonketotic diabetes at birth, frequently alternating with hypoglycemia (2). Fasting insulin concentrations >25 μUI/mL (>150 pmolL/L) are considered to be a sign of SIR in lean children/adolescents (1), but in neonates with c.SIR insulin levels in the triple/quadruple-digit range (>100-1000 μU/mL) (2) are often observed at random sampling (neonates are fed every 2-4 hours), along with high C-peptide levels. c.SIR is caused by biallelic, usually recessive, variants in genes that impair insulin action like *INSR* (2, 3).

NDM is not frequent, with an estimated incidence in Europe of about 1:100 000 live births (4), while c.SIR is considered exceedingly rare (an estimated 1:4 000 000 live births for Donohue and Rabson–Mendenhall syndrome) (2). According to the International Society for Pediatric and Adolescent guidelines 2022, all cases with a clinical diagnosis of NDM and c.SIR deserve genetic testing (1).

Until 2013, a relatively short list of genes/chromosomal defects was used to investigate NDM and c.SIR in Europe and the United States, namely, *KCNJ11*, *INS*, *ABCC8* (both permanent NDM and TNDM), 6q24 (TNDM), and *INSR* (c.SIR), while some rare, recessive permanent NDM (PNDM) genes (eg, *EIF2AK3*, *GCK*, *PDX1*, *PTF1A*, *GLIS3*, others) were screened “on demand,” according to the proband's phenotype. By screening the 4 main genes in 27 subjects with NDM identified between 2005 and 2010, we were able to reach a genetic diagnosis in 18 (66%) (4). In the last 15 years, however, the list of genes causing nonautoimmune PNDM has been constantly increasing and now exceeds 25. New genes of autoimmune PNDM have been also discovered, adding to the prototype of this subgroup (ie, *FOXP3*) (1). In contrast, just 3 genetic defects (6q24, *ABCC8*, *KCNJ11*) still account for about 90% of cases with TNDM (1, 5). Nevertheless, a few new TNDM genes have been found in recent years (1).

In the last decade, new techniques of DNA sequencing, collectively called “next-generation sequencing” or NGS, progressively replaced the Sanger method in all fields of genetics, including NDM (6, 7). By allowing parallel massive DNA sequencing of thousands genes, NGS has proven to be the ideal method to investigate diseases with high genetic heterogeneity, accelerating the identification of pathogenic variants in rare loci.

In this paper we evaluated the impact of NGS on NDM and c.SIR by comparing the 2003-2012 period, when Sanger was the only method in use in Italy, and 2013-2022 period, when NGS progressively became the method of choice.

## Materials and Methods

We utilized the definition of NDM and c.SIR described earlier and in (1). NDM was diagnosed in a patient presenting with plasma glucose in the diabetes range within 6 months of age, along with low/undetectable insulin/C-peptide levels. c.SIR was suspected in neonates with diabetes, high insulin (>25 μU/mL), and/or C-peptide levels.

Sanger DNA sequencing of frequent NDM/c.SIR genes (*KCNJ11*, *INS*, *ABCC8*, and *INSR*) and methods utilized to investigate 6q24 defects (TNDM) were performed as previously described (5, 8, 9). For NGS, clinical exome sequencing (Twist Custom Panel kit, 8245 genes; Twist Bioscience, San Francisco, CA, USA) was performed at Bambino Gesù Children’s Hospital on a NovaSeq6000 platform (Illumina, San Diego, CA, USA).

The following genes were filtered out for analysis: nonautoimmune NDM: *ABCC8*, *CISD2*, *CNOT1*, *EIF2B1*, *EIF2S3*, *EIF2AK3*, *GATA4*, *GATA6*, *GCK*, *GLIS3*, *HNF1B*, *IER3IP1*, *INS*, *KCNJ11*, *KCNMA1*, *MNX1*, *NEUROD1*, *NEUROG3*, *NKX2-2*, *PAX6*, *PDX1*, *PTF1A*, *RFX6*, *SLC2A2*, *SLC19A2*, *WFS1*, *YIPF5*, and *ZFP57*; when necessary, sequencing of *INS* promoter (both for PNDM and TNDM) and *PTF1A* distal enhancer (cases with suspected pancreas agenesis with no extrapancreatic features) were performed; in addition *ONECUT1*, a recently identified gene was examined in 2 cases identified during 2013-2022 and negative to the initial screening; autoimmune NDM: *CTLA4*, *FOXP3*, *IL2RA*, *ITCH*, *LRBA*, and *STAT3*; congenital SIR: *AGPAT2*, *BSCL2*, *CAV1*, *CAVIN1*, and *INSR.* Other Italian laboratories involved in NDM/c.SIR genetic screening based in Milan (San Raffaele Hospital), Naples (CEINGE), San Giovanni Rotondo (IRCCS Casa Sollievo della Sofferenza), and Novara (University of Eastern Piedmont) utilized slightly different NGS methods and lists of genes.

### Statistics

Count and rate have been reported for each group. Chi-square analysis was used to compare the subgroups. Statistical significance was set at *P* < .05.

Informed consent to perform genetic studies for diagnostic purpose was obtained by parents or guardians of the probands. Ethics committee approval was not requested, since the General Authorization to Process Personal Data for Scientific Research Purposes (authorization no. 9/2014) declared that retrospective archive studies that use identifier codes, preventing the data from being traced back directly to the data subject, do not need ethics approval.

## Results

During 2003-2022 we observed 96 cases with NDM (PNDM: 45; TNDM: 51). Fifty probands were identified between 2003 and 2012 (PNDM: 26; TNDM: 24) and 46 between 2013 and 2022 (PNDM: 19; TNDM: 27). c.SIR cases were 5 during 2003-2012 and 3 between 2013 and 2022.

### Permanent NDM

**Figure 1.**
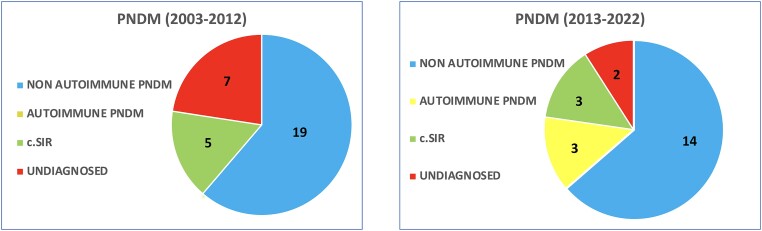
Genetic etiology of PNDM in the period 2003-2012 compared with the period 2013-2022.

Genetic etiology of PNDM was established in 19 individuals (73%) from 2003-2012 and in 17 cases (89.5%) from 2013-2022 (Fig. 1). The main cause of PNDM in both periods was *KCNJ11* (20 patients) followed by *INS* (8 patients) (Table 1). *KCNJ11*, *ABCC8*, and *INS* variants identified have been previously described by our group or by others (8, 10-13) (Table 2). All but 1 persons with PNDM caused by *KCNJ11* or *ABCC8* pathogenic variants were successfully switched from insulin to sulfonylureas (Table 2).

**Table 1.** Genetics causes of PNDM in Italy 2003-2022

	2003-2012 no. of cases per locus	2013-2022 no. of cases per locus	Consanguinity of parents
*KCNJ11*	12	8	n.a.
*INS*	6	2	n.a.
*ABCC8*	—	1	n.a.
*GATA4*	1	—	n.a.
*GATA6*	—	1	n.a.
*PDX1*	—	1	no
*RFX6*	—	1	yes
*FOXP3*	—	1	n.a.
*IL2RA*	—	1	?
*LRBA*	—	1	no
Diagnosed	19 (73%)	17 (89.5%)	1
Unknown	7	2	2
Total	26	19	3

Comparison of % of case diagnosed 2003-2012 vs 2013-2022. *P* = n.s.

Comparison of % of rare forms of PNDM 2003-2012 (1 out 19; 5.2%) vs 2013-2022 (6 out 17; 35.2%): *P* = .023. Rare genes/forms: *GATA4*, *GATA6*, *PDX1*, *RFX6*, *FOXP3*, *IL2RA*, *LRBA*.

Abbreviations: ?, unknown; n.a., not applicable; PNDM, permanent neonatal diabetes mellitus.

**Table 2.** Variants identified in common PNDM/TNDM genes *KCNJ11*, *INS*, and *ABCC8*

	*KCNJ11*	*INS*	*ABCC8*
PNDM	His46Tyr, Gly53Val, Val59Ala, Val59Met (2), Arg201Ser, Arg201Cys (4), Arg201His (6), Glu322Lys, Tyr330His*^a^*	Leu30Pro, Leu30Val, Gly32Ser, Tyr50Cys, Arg89Cys (2), Cys96Tyr, Leu105Pro	Glu1141Lys
TNDM	Cys42Arg, Arg50Gln (4), Glu179Lys, Ser225Arg*^b^*, Glu227Leu, Glu229Lys (2), Thr293Ser		Leu213Pro, Leu225Pro*^b^*, Ser459Arg, Thr540Ile*^b^*+4415-13G>A, Arg825Trp, Arg1182Gln, Arg1380Cys (3) Arg1380His (2) Val1523Met

Abbreviations: PNDM, permanent neonatal diabetes mellitus; TNDM, neonatal diabetes mellitus transient subtype.

^*a*^Not responsive to sulfonylureas.

^*b*^Novel variants.

Between 2003 and 2012 a single neonate with PNDM associated with a rare gene (*GATA4*) was identified (14) (Table 1). In contrast, during 2013-2022 we identified 3 infants with rare syndromic PNDM caused by genes encoding for transcription factors *RFX6* (homozygous) (15), *PDX1* (compound heterozygous) (16), *GATA6* (NM_005257.6: c.1502C>G; p.Ser501Ter, heterozygous, novel variant), and 3 cases with autoimmune PNDM caused by *FOXP3* (17), LRBA (compound heterozygous) (18), and the novel homozygous variant c.227G>A; p.Trp76Ter of *IL2RA* gene (NM_000417.2) (Fig. 1, Table 1 and Table 3). Rare forms of PNDM, either dominant, recessive, or X-linked were identified more frequently in 2013-2022 than 2003-2012 (*P* = .023) (Table 1). Autoimmune PNDM was genetically diagnosed in 15.8% of neonates (3 out of 19) during 2013 and in none between 2003 and 2012.

**Table 3.** Clinical features of FOXP3, IL2RA, and LRBA autoimmune permanent neonatal diabetes mellitus cases (2012-2022)

	FOXP3	IL2RA	LRBA
Age at onset	Day 1	Day 8	6 months
Blood glucose, mg/dL	400	1504 (severe DKA)	700 (DKA)
C-peptide	<0.3 ng/mL	n.a.	n.a.
Type 1 diabetes autoantibodies	ICA	ICA	IA-2A, IAA
Other autoantibodies	Antiharmonin, antivillin	No	No
Persistent diarrhea	Yes at 1 month	No	Yes at 9 months
Bone marrow transplant	Yes	Yes (at 5 m)	Yes (at 16 months)
Birth weight	2000	3380	3950

Abbreviations: DKA, diabetic ketoacidosis; n.a., not available.

No genetic diagnosis was obtained in 7 infants with PNDM during 2003-2012 (27%) and in 2 during 2013-2022 (10.5%) (Fig. 1 and Table 1). Six out of 9 were syndromic (4 diagnosed during 2003-2012): 1 with IPEX-like features (*FOXP3*, *NEUROG3* negative, deceased), 1 with heart malformation and clinical and laboratory findings compatible with pancreas and gallbladder agenesis (*PDX1*, *GATA6* negative, deceased), 1 with severe prematurity and multicystic kidney (lost at follow-up), 1 with cleidocranial dysplasia (lost at follow up), 1 with severe epilepsy not responsive to drugs (deceased), 1 with intestinal malformation carrying a heterozygous *RFX6* missense variant (data not shown). Among the 3 subjects with nonsyndromic PNDM without a genetic diagnosis, 1 carried a *ONECUT1* variant in heterozygous state (19).

### NDM transient subtype

**Figure 2.**
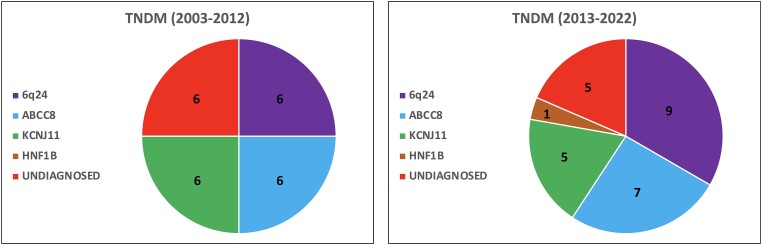
Genetic etiology of TNDM in the period 2003-2012 compared with the period 2013-2022.

A genetic diagnosis was reached in 18 (75%) and 22 (81.5%) infants with TNDM during 2003-2012 and 2013-2022, respectively (Fig. 2). During 2003-2012 pathogenic variants in *ABCC8*, *KCNJ11*, and 6q24 aberrations were identified in 6, 6, and 6 cases (Table 4) (5). All 6 cases without a genetic diagnosis were lost to follow-up. Four *ABCC8*/*KCNJ11*-negative subjects did not perform a 6q24 test and 1 did not perform any genetic test. Another neonate with heart and kidney malformation was negative for 6q24 but DNA sequencing of other TNDM genes was not performed.

**Table 4.** Genetic causes of neonatal diabetes mellitus transient subtype in Italy 2003-2022

	2003-2012 no. of cases per locus	2013–2022 no. of cases per locus
*KCNJ11*	6	5
*ABCC8*	6	7
6q24	6	9
*HNF1B*	—	1
Diagnosed	18	22
Unknown/incomplete	6	5
Total	24	27

In the period 2013-2022 we identified 9 probands with 6q24 defects, 7 and 5 with pathogenic variants in *ABCC8* and *KCNJ11*, and 1 with a rare *HNF1B* mutation (NM_000458.4:c.1045+1G>A, p. ?) (20) (Fig. 2 and Table 4). Most of the *ABCC8-* and *KCNJ11-*TNDM pathogenic or likely pathogenic variants identified during the whole period 2003-2022 have been previously described, while 2 were novel. A likely pathogenic *KCNJ11* de novo variant (NM_000525.4: c.675C>A, p.Ser225Arg) (Table 2) was identified in a patient who remitted to normal glycemia without any insulin therapy 1 week after diabetes onset. In 2 brothers with TNDM diagnosed with diabetes in 2010 (first observation period) and 2017 (second observation period) compound heterozygous variants of *ABCC8* (NM_000352.6: c.4412-13G>A, p. ?; c.1619C>T, p.Thr540Ile) (Table 2) were detected. The variant c.4412-13G>A, maternally inherited, was functionally tested by others (21) (where the variant is denoted as c.4415-13G>A). This study showed that c.4412-13G>A impacts on splicing by inducing retention of 11 nucleotides from intron 37 and also a new 3' splice site within the intron. Thus, we classified the variant as pathogenic, loss of function (American College of Medical Genetics and Genomics, class 5) (22). The paternally inherited variant Thr540Ile (previously identified by Sanger) is not present in gnomAD database and has been considered to be likely pathogenic per American College of Medical Genetics and Genomics rules (22) because it is in trans with a pathogenic variant of the same gene. Both parents of these 2 patients were normoglycemic at the oral glucose tolerance test.

In 2 out of 6 patients with TNDM negative for 6q24 from 2013-2022, *KCNJ11* and the rare TNDM genes *SLC2A2* and *HNF1B*, an *ABCC8* variant of uncertain significance was identified. In 1 patient the variant of uncertain significance was spontaneous (c.2959T>C, p.Ser987Pro), while in the other (c.157A>T, p.Ser53Cys) it was inherited from the normoglycemic mother. These 2 subjects were included in the TNDM group of unknown etiology (Fig. 2). The remaining 4 infants with TNDM (2013-2022) were negative for *ABCC8* and *KCNJ11* and lost to follow-up without being tested for 6q24.

### NDM and Mode of Inheritance

Most variants in frequent NDM genes (*KCNJ11*, *INS*, *ABCC8*) are dominant and in many patients occur spontaneously (Table 5, second column from the left); however, familial cases with typical vertical transmission are not infrequent (Table 5, third column). Interestingly, in patients with TNDM due to *KCNJ11* or *ABCC8* mutations, it is not rare to observe a different age at diabetes onset (adolescence, adulthood, ie, not neonatal) in the parent carrying the variant ((6), and this investigation). As already described by others (6), recessive PNDM forms (Table 5) are rarely identified in patients born to nonconsanguineous parents. In our dataset, most individuals with recessive forms of NDM carried homozygous variants, but 2 individuals bore compound heterozygous variants of *PDX1* (pancreas hypoplasia) and *LRBA* (autoimmune case PNDM).

**Table 5.** Mode of inheritance of variants identified in patients with PNDM, TNDM, and c.SIR

Gene	De novo, autosomal dominant	Autosomal dominant	Autosomal recessive	X-linked
*KCNJ11-PNDM*	19	1		
*KCNJ11-TNDM*	4	7	1	
*ABCC8-PNDM*		1		
*ABCC8-TNDM*	7	4	2	
*INS-PNDM*	7	1		
*GATA4-PNDM*	1			
*GATA6-PNDM*	1			
*RFX6-PNDM*			1	
*PDX1-PNDM*			1	
*HNF1B-TNDM*	1			
*LRBA-PNDM*			1	
*IL2RA-PNDM*			1	
*FOXP3*				1
*INSR*			7	
*BSCL2*			1	

Please note that 6q24 cases have not been reported in the table. Aberrations of 6q24 are usually sporadic, but some specific defects can be inherited. All patients with PNDM or TNDM caused by heterozygous, spontaneous mutations (de novo) have a 50% chance of passing the mutant allele to each child. In this table, 14 cases inherited their variant from a parent; in 2 ABCC8 kindreds, however, the parent carrying the variant was normoglycemic, ie, nonpenetrance was observed.

Abbreviations: c.SIR, congenital severe insulin resistance; PNDM, permanent neonatal diabetes mellitus; TNDM, NDM transient subtype.

### NDM and Prematurity

When diagnosing NDM, prematurity (<37 weeks of gestational age) may pose a challenge because premature neonates can present with transient hyperglycemia (23). Among infants with established genetic diagnosis during the whole period of observation (2003-2022), 3 PNDM (out of 36, 8.3%) and 6 TNDM (out of 39, 15.3%) were premature. Among neonates with TNDM, the most frequent genetic findings were 6q24 and *ABCC8* (Table 6). However, prematurity was more frequent in patients (PNDM + TNDM, 2003-2022) without diagnosis (8 out of 21 or 38%) compared with those with a genetic diagnosis (9 out of 75, or 12%) (*P* = .005625). Notably in 3 out of 4 premature patients with TNDM, 6q24 testing was not performed, while all 4 premature PNDM without a genetic diagnosis (born between 2008 and 2012) were syndromic.

**Table 6.** Premature (<37 weeks of gestational age) per locus

	PNDM 2003-2022 no. of cases per locus	PNDM 2003-2022 no. premature (<37 weeks)	TNDM 2003-2022 no. of cases per locus	TNDM 2003-2022 no. premature (<37 weeks)
KCNJ11	20	1	10	1
INS	8	/	/	/
ABCC8	1	/	13	2
6q24	/	/	15	2
GATA4	1	/	/	/
GATA6	1	1	/	/
PDX1	1	1	/	/
RFX6	1	/	/	/
HNF1B	/	/	1	1
FOXP3	1	/	/	/
IL2RA	1		/	/
LRBA	1	/	/	/
Diagnosed	36	3 (8.3%)	39	6 (15.3%)
Unknown	9	4 (44.4%)	12	4 (33.3%)
Total	45	7	51	10

2003-2022 overall: diagnosed premature = 12%; unknown premature = 38%, *P* = .005625.

Abbreviations: PNDM, permanent neonatal diabetes mellitus; TNDM, NDM transient subtype.

### Congenital SIR

Five neonates with biallelic *INSR* variants were diagnosed with c.SIR in the period 2003-2012 (9). During 2013-2022, 2 apparently unrelated cases with c.SIR associated with the same homozygous *INSR* mutation (NM_000208.4; c.3289C>T; p.Gln1097Ter) and 1 with the novel homozygous *BSCL2* variant (NM_001122955.4: c.832-833delGA; p.Asn278GlnfsTer18) (Table 7) were identified.

**Table 7.** Clinical features of *INSR* and *BSCL2* cases (2003-2022)

	*INSR*-6	*INSR*-7	*BSCL2*	*INSR*-1 to 5 (2003-2012)
Insulin, μU/mL (min/max)	241/2980	553	61/84.8	54/3950
Blood glucose mg/dL (min/max)	30-320 (CGM)	undetectable-327	46-480	<30/621
Triglycerides (mg/dL)	83	n.a.	1180	115, 126, 182 (n.a. in 2 pat.)
Plasma K+	Low	n.a.	Normal	Low (3 out of 5)
Hirsutism	Yes	Yes	Yes	Yes (4 out of 5)
Protruding abdomen	Yes	Yes	Yes	Yes (4 out of 5)
Abdominal ultrasound	Nephrocalcinosis	Nephrocalcinosis	Hepatomegaly	Nephrocalcinosis: 2 out of 5
Aldosterone/renin	High/high	n.d.	n.d.	high/high (2 out of 5)
Birth weight	1840	1149	3300	990-2225
Deceased <2 years of age	No	Yes	No	3 out of 5
Consanguinity of parents	Yes	Yes	Yes	1 out of 5

Abbreviations: CGM, continuous glucose monitoring; n.a., not available.

Six out of 7 *INSR* patients (2003-2022) had classical c.SIR features (intrauterine growth restriction, hirsutism, acanthosis nigricans, facial dysmorphisms) and could be divided in Donohue syndrome (4 patients) and Rabson–Mendenhall syndrome (3 patients) according to survival (4 patients with Donohue syndrome died before 24 months of age). Only in a patient with Rabson–Mendenhall, now 14 years old, hirsutism and acanthosis nigricans were not evident at birth, but later in life. Moreover, 2 *INSR* patients of the period 2013-2022 presented with nephrocalcinosis, a specific feature of *INSR*-c.SIR (Table 7) (9). The single case with the *BSCL2* variant somehow resembled *INSR*-c.SIR because the case presented with hyperglycemia with episodes of hypoglycemia, hirsutism, protruding abdomen, and lipodystrophy, but differed for birth weight (normal) and triglyceride levels (very high in *BSCL2*, normal–low in *INSR*; Table 7) and absence of nephrocalcinosis.

Precise genetic diagnosis impacted on therapy of individuals with c.SIR: the patient carrying the *BSCL2* variant was started on metreleptin (24) with excellent results for glucose and lipid metabolism (manuscript in preparation). In 2 patients with Rabson–Mendenhall syndrome (9) with diabetes of long duration (>3 years) and modest or null response to metformin and insulin therapy, the SGLT2i empagliflozin (5 mg) was introduced as an add-on treatment with good (25) to excellent results (manuscript in preparation). All patients with congenital SIR reported in this investigation bear biallelic, recessive mutations (Table 7).

### NDM and c.SIR Incidence

**Figure 3.**
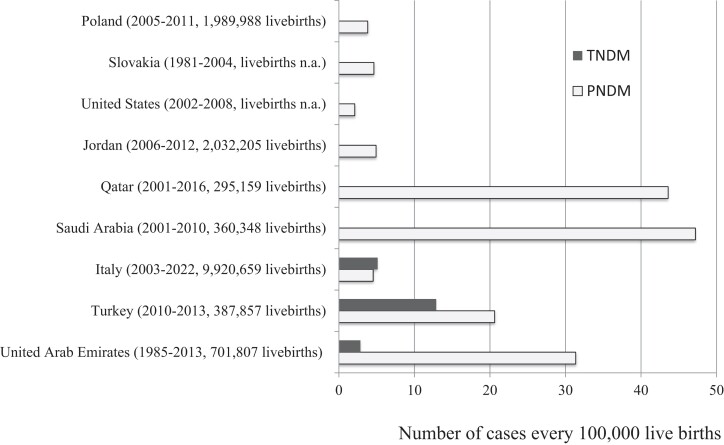
Estimated incidence of NDM in different countries. References concerning incidence in individual countries: Poland (28), Slovakia (29), United States (30), Jordan (31), Qatar (32), Saudi Arabia (33), Turkey (34), United Arab Emirates (35).

During 2003-2012, there were 5 469 539 live births in Italy, setting the incidence of NDM to 1:109 390 (or 0.88/100 000; 95% CI 0.59-1.16), while in the decade 2013-2022 there were 4 451 120 live births and the incidence was 1:96 763 (or 1.05/100 000; 95% CI 0.68-1.42). For the whole period 2003-2022 the incidence was 1:103 340, well in agreement with our previous estimates (4). Of interest, PNDM incidence in Europe is confirmed to be much lower than Middle Eastern countries, while for TNDM the effect of consanguinity is less evident (Fig. 3) (26-33). The incidence of c.SIR during the entire period 2003-2022 was 1:1 240 082 live births.

## Discussion

Results of our comparison between 2 decades of investigation on genetic causes of NDM and congenital SIR in Italy shows that NGS made a difference. Pathogenic variants in frequent NDM genes (*KCNJ11*, *INS*, *ABCC8*, 6q24) accounted for 72% and 69.5% in the 2 periods of observation, a similar figure to our previous finding (4). In contrast, during the period 2013-2022, when NGS became increasingly available in genetic laboratories, we identified pathogenic variants in rare recessive or X-linked PNDM genes such as *RFX6*, *PDX1*, *LRBA*, *IL2RA*, and *FOXP3*, and a dominant *GATA6* variant in a case with pancreas agenesis and heart malformation. In addition, rare causes of TNDM and c.SIR (ie, *HNF1B* and *BSCL2*, respectively) were readily detected. These results cannot be attributed to newly identified NDM or c.SIR genes, because 6 of those involved in this study were discovered when Sanger sequencing was the only method used to sequence DNA in Italy, namely, *PDX1* (identified in 1997), *FOXP3* (2001), *BSCL2* (2001), *HNF1B* (2006 as cause of pancreas hypoplasia), *IL2RA* (previously known as CD25, 2007), *RFX6* (2009), and *GATA6* (2012) and only 1, namely, *LRBA*, has been identified in recent years (2017) as a cause of autoimmune PNDM. In 2 subjects, 1 with isolated diabetes (19), NGS revealed heterozygous variants in 2 recessive genes known to cause syndromic PNDM (*RFX6*, *ONECUT1*) (34, 35). These 2 cases have been considered unsolved and await further investigation to define the etiology of neonatal diabetes.

NGS, publicly available database (eg, gnomAD), and functional studies were instrumental to identify and classify pathogenic/likely pathogenic *ABCC8* variants in 2 brothers with TNDM that have not been previously resolved. The 2 brothers carried a loss of function variant in 1 allele and the missense variant Thr540Ile in the other. We reasoned that Thr540Ile has a mild activating function and causes TNDM because it is functionally hemizygous. This mechanism of disease has been previously demonstrated in *ABCC8*-PNDM (36), but to the best of our knowledge, it has never been described in TNDM.

During 2013-2022, we observed an apparent increase in TNDM, likely because screening of blood glucose at birth has become more common with better detection of mild, transient diabetes (5). Nevertheless, many TNDM cases remained unsolved, probably because of a lesser “incentive” to pursue genetic testing in nonsyndromic patients with modest, remitting hyperglycemia treated with short-lived insulin therapy or only rehydration. Of interest, a patient with *KCNJ11*-TNDM with diabetes onset in 2022 entered remission without any insulin or sulfonylurea treatment, confirming an observation we made in 2 other patients with TNDM associated with *KCNJ11* pathogenic variants (5). Therefore, caution should be exerted with the implementation of sulfonylurea therapy before the results of genetic testing because of the risk of hypoglycemia. In addition, this finding reinforces the notion that insulin treatment is an applicable but in some instances dispensable criterion for the diagnosis of TNDM.

NGS was especially useful to identify rare genes of autoimmune PNDM and c.SIR. For instance, patients with *FOXP3*, *IL2RA*, and *LRBA* PNDM may present similar extrapancreatic features (eg, enteropathies, hypothyroidism) (37). In addition, *LRBA* can cause autoimmune PNDM without syndromic symptoms/signs associated with severe immune dysregulation (38). Simultaneous screening allowed swift identification of autoimmune PNDM genes involved, that in turn lead to a specific therapy (ie, bone marrow transplant). In addition, quick identification of pathogenic variants in *RFX6* (34) and *PDX1* (previously known as *IPF1*) (39) along with signs of exocrine insufficiency in PNDM cases with probable pancreatic hypoplasia/agenesis prompted replacement therapy with pancreatic enzymes. Importantly, this intervention was not based on ultrasound imaging of the pancreas.

Our data confirm that *INSR* mutations are a major cause of c.SIR, with NGS identifying a single patient with the *BSCL2* pathogenic variant. In this case, physical examination was puzzling, but 2 clinical features set apart the infant bearing an *BSCL2* variant from the *INSR* group: lack of intrauterine growth restriction and hypertriglyceridemia (Table 7) (40, 41). Finally, we found that c.SIR is 12-fold rarer than NDM but about 3 times more frequent than a previous estimate (2). Considering that major causes of NDM in Italy are autosomal dominant genes (*KCNJ11*, *INS*, and most of *ABCC8* cases) while c.SIR is recessive, this is not an unexpected finding.

A limitation of our study is the small number of patients involved, which does not allow robust conclusions to be drawn about the incidence of rare, recessive forms of PNDM that are common in the Middle East (Fig. 3). For instance, in Abu Dhabi, Turkey, and Quatar biallelic pathogenic variants in the *PTF1A* enhancer, *INS* promoter, and *EIF2AK3* genes account for 30% to 78% of all cases of PNDM (30, 33, 42), although we did not identify any such variant. Still, our study illustrates the advantages provided by NGS over Sanger sequencing in terms of time to and precision of genetic diagnosis. We conclude that combining results of massive parallel sequencing with pertinent clinical features of the proband allow prompt confirmation of genetic findings and timely administration of the best available treatments.

## Data Availability

Original data generated and analyzed during this study are included in this published article or in the data repositories listed in References.

## Abbreviations

c.SIR: congenital severe insulin resistance
DKA: diabetic ketoacidosis
NDM: neonatal diabetes mellitus
NGS: next-generation sequencing
PNDM: permanent NDM
TNDM: transient NDM

## References

[1] Greeley SAW , Polak M, Njølstad PR, et al ISPAD clinical practice consensus guidelines 2022: the diagnosis and management of monogenic diabetes in children and adolescents. Pediatr Diabet. 2022;23(8):1188‐1211. 10.1111/pedi.13426 PMC10107883 36537518

[2] Taylor SI , Cama A, Accili D, et al Mutations in the insulin receptor gene. Endocr Rev. 1992;13(3):566‐595. 1330507 10.1210/edrv-13-3-566

[3] Patni N , Garg A. Lipodystrophy for the diabetologist—what to look for. Curr Diab Rep. 2022;22(9):461‐470. 35821558 10.1007/s11892-022-01485-w PMC10704567

[4] Iafusco D , Massa O, Pasquino B, et al Minimal incidence of neonatal/infancy onset diabetes in Italy is 1:90,000 live births. Acta Diabetol. 2012;49(5):405‐408. 21953423 10.1007/s00592-011-0331-8 PMC3464369

[5] Bonfanti R , Iafusco D, Rabbone I, et al Differences between transient neonatal diabetes mellitus subtypes can guide diagnosis and therapy. Eur J Endocrinol. 2021;184(4):575‐585. 33606663 10.1530/EJE-20-1030

[6] De Franco E , Flanagan SE, Houghton JAL, et al The effect of early, comprehensive genomic testing on clinical care in neonatal diabetes: an international cohort study. Lancet. 2015;386(9997):957‐963. 26231457 10.1016/S0140-6736(15)60098-8 PMC4772451

[7] Letourneau LR , Greeley SAW. Congenital diabetes: comprehensive genetic testing allows for improved diagnosis and treatment of diabetes and other associated features. Curr Diab Rep. 2018;18(7):46. 29896650 10.1007/s11892-018-1016-2 PMC6341981

[8] Russo L , Iafusco D, Brescianini S, et al Permanent diabetes during the first year of life: multiple gene screening in 54 patients. Diabetologia. 2011;54(7):1693‐1701. 21544516 10.1007/s00125-011-2094-8 PMC3110270

[9] Grasso V , Colombo C, Favalli V, et al Six cases with severe insulin resistance (SIR) associated with mutations of insulin receptor: is a Bartter-like syndrome a feature of congenital SIR? Acta Diabetol. 2013;50(6):951‐957. 23824322 10.1007/s00592-013-0490-x

[10] Massa O , Iafusco D, D’Amato E, et al *KCNJ11* activating mutations in Italian patients with permanent neonatal diabetes. Hum Mutat. 2005;25(1):22-27. 15580558 10.1002/humu.20124

[11] McClenaghan C , Rapini N, De Rose UD, et al Sulfonylurea-insensitive permanent neonatal diabetes caused by a severe gain-of-function Tyr330His substitution in Kir6.2. Horm Res Paediatr. 2022;95(3):215‐223. 34999583 10.1159/000521858 PMC9259755

[12] Colombo C , Porzio O, Liu M, et al Seven mutations in the human insulin gene linked to permanent neonatal/infancy-onset diabetes mellitus. J Clin Invest. 2008;118(6):2148‐2156. 18451997 10.1172/JCI33777 PMC2350430

[13] Ortolani F , Piccinno E, Grasso V, et al Diabetes associated with dominant insulin gene mutations: outcome of 24-month, sensor-augmented insulin pump treatment. Acta Diabetol. 2016;53(3):499‐501. 26239141 10.1007/s00592-015-0793-1 PMC4877418

[14] D'Amato E , Giacopelli F, Giannattasio A, et al Genetic investigation in an Italian child with an unusual association of atrial septal defect, attributable to a new familial *GATA4* gene mutation, and neonatal diabetes due to pancreatic agenesis. Diabet Med. 2010;27(10):1195-1200. 20854389 10.1111/j.1464-5491.2010.03046.x

[15] Calcaterra V , Chiricosta L, Mazzon E, et al Determining oncogenic patterns and cancer predisposition through the transcriptomic profile in Mitchell-Riley syndrome wth heteroplastic gastric mucosa and duodenal atesia: a case report. Orphanet J Rare Dis. 2021;16(1):455. 34715892 10.1186/s13023-021-02093-9 PMC8556982

[16] Rapini N , Patera IP, Schiaffini R, et al Monogenic diabetes clinic (MDC): 3-year experience. Acta Diabetol. 2023;60(1):61‐70. 36178555 10.1007/s00592-022-01972-2 PMC9813184

[17] Romano F , Tinti D, Spada M, Barzaghi F, Rabbone I. Neonatal diabetes in a patient with IPEX syndrome: an attempt at balancing insulin therapy. Acta Diabetol. 2017;54(12):1139‐1141. 28988367 10.1007/s00592-017-1057-z

[18] Galati A , Muciaccia R, Marucci A, et al Early-onset diabetes in an infant with a novel frameshift mutation in *LRBA*. Int J Environ Res Public Health. 2022;19(17):11031. 36078750 10.3390/ijerph191711031 PMC9517908

[19] Prudente S , Andreozzi F, Mercuri L, et al Contribution of *ONECUT1* variants to different forms of non-autoimmune diabetes mellitus in Italian patients. Acta Diabetol. 2022;59(8):1113‐1116. 35482136 10.1007/s00592-022-01889-w

[20] Pezzino G , Ruta R, Rapini N, et al A rare cause of transient neonatal diabetes mellitus (TNDM): spontaneous *HNF1B* splice variant. Diabet Med. 2024;41(2):e15202. 37597176 10.1111/dme.15202

[21] Saint-Martin C , Cauchois-Le Mière M, Rex E, et al Functional characterization of *ABCC8* variants of unknown significance based on bioinformatics predictions, splicing assays, and protein analyses: benefits for the accurate diagnosis of congenital hyperinsulinism. Hum Mutat. 2021;42(4):408‐420. 33410562 10.1002/humu.24164 PMC8049974

[22] Richards S , Nazneen A, Bale S, et al Standards and guidelines for the interpretation of sequence variants: a joint consensus recommendation of the American college of medical genetics and genomics and the association for molecular pathology. Genet Med. 2015;17(5):405‐424. 25741868 10.1038/gim.2015.30 PMC4544753

[23] Besser REJ , Flanagan SE, Mackay JG, et al Prematurity should not prevent genetic testing for neonatal diabetes. Pediatrics. 2016;138(3):e20153926. 27540106 10.1542/peds.2015-3926 PMC5049686

[24] Meehan CA , Cochran E, Kassai A, Brown RJ, Gorden P. Metreleptin for injection to treat the complications of leptin deficiency in patients with congenital or acquired generalized lipodystrophy. Expert Rev Clin Pharmacol. 2016;9(1):59‐68. 26465174 10.1586/17512433.2016.1096772 PMC4931926

[25] Galderisi A , Tamborlane W, Taylor SI, Attia N, Moretti C, Barbetti F. SGLT2i improve glycemic control in patients with congenital severe insulin resistance. Pediatrics. 2022;150(1):e2021055671. 35652305 10.1542/peds.2021-055671

[26] Fendler W , Borowiec M, Baranowska-Jazwiecka A, et al Prevalence of monogenic diabetes amongst Polish children after a nationwide genetic screening campaign. Diabetologia. 2012;55(10):2631‐2635. 22782286 10.1007/s00125-012-2621-2 PMC3433657

[27] Stanik J , Gasperikova D, Paskopva M, et al Prevalence of permanent neonatal diabetes in Slovakia and successful replacement of insulin with sulphonylurea therapy in *KCNJ11* and *ABCC8* mutation carriers. J Clin Endocrinol Metab. 2007;92(4):1276-1282. 17213273 10.1210/jc.2006-2490 PMC7611849

[28] Stoy J , Greeley SA, Paz VP, et al Diagnosis and treatment of neonatale diabetes: a United States experience. Pediatr Diabetes. 2008;9(5):450‐459. 18662362 10.1111/j.1399-5448.2008.00433.x PMC2574846

[29] Abujbara MA , Liswi MI, El-Khateeb MS, Flanagan SE, Ellard S, Ajlouni KM. Permanent neonatal diabetes mellitus in Jordan. J Pediatr Endocrinol Metab. 2014;27(9-10):879‐883. 24825091 10.1515/jpem-2014-0069

[30] Al-Khawaga S , Mohammed I, Saraswathi S, et al The clinical and genetic characteristics of permanent neonatal diabetes (PNDM) in the state of Qatar. Mol Genet Genomic Med. 2019;7(10):e00753. 31441606 10.1002/mgg3.753 PMC6785445

[31] Habeb AM , Al-Magamsi MS, Eid IM, et al Incidence, genetics, and clinical phenotype of permanent neonatal diabetes mellitus in Northwest Saudi Arabia. Pediatr Diabetes. 2012;13(6):499‐505. 22060631 10.1111/j.1399-5448.2011.00828.x

[32] Demirbilek H , Arya VB, Ozbek MN, et al Clinical characteristics and molecular genetic analysis of 22 patients with neonatal diabetes from the South-Eastern region of Turkey: predominance of non-KATP channel mutations. Eur J Endocrinol. 2015;172(6):697‐705. 25755231 10.1530/EJE-14-0852 PMC4411707

[33] Deeb A , Habeb A, Kaplan W, et al Genetic characteristics, clinical spectrum, and incidence of neonatal diabetes in the Emirate of AbuDhabi, Unted Arab Emirates. Am J Med Genet. 2016;170(3):602‐609. 26463504 10.1002/ajmg.a.37419

[34] Smith SB , Qu HQ, Taleb N, et al Rfx6 directs islet formation and insulin production in mice and humans. Nature. 2010;463(7282):775‐780. 20148032 10.1038/nature08748 PMC2896718

[35] Philippi A , Heller S, Costa IG, et al Mutations and variants of *ONECUT1* in diabetes. Nat Med. 2021;27(11):1928-1940. 34663987 10.1038/s41591-021-01502-7 PMC9356324

[36] Ellard S , Flanagan SE, Girard CA, et al Permanent neonatal diabetes caused by dominant, recessive, or compound heterozygous SUR1 mutations with opposite functional effects. Am J Hum Genet. 2007;81(2):375‐382. 17668386 10.1086/519174 PMC1950816

[37] Johnson MB , Hattersley AT, Flanagan S. Monogenic autoimmune disease of the endocrine system. Lancet Diabetes Endocrinol. 2016;4(10):862‐872. 27474216 10.1016/S2213-8587(16)30095-X

[38] Sanyoura M , Lundgrin EL, Subramanian HP, et al Novel compound heterozygous *LRBA* dletions in a 6-month-old with neonatal diabetes. Diab Res Clin Pract. 2021;175:108798. 10.1016/j.diabres.2021.108798 PMC11056189 33845048

[39] Stoffers DA , Zinkin NT, Stanojevic V, Clarke WL, Habener JF. Pancreatic agenesis attributable to a single nucleotide deletion in the human *IPF1* gene coding sequence. Nat Genet. 1997;15(1):106-110. 8988180 10.1038/ng0197-106

[40] Friguls B , Coroleu W, del Alcazar R, Hilbert P, Van Maldergem L, Pintos-Morell G. Severe cardiac phenotype of Berardinelli-Seip congenital lipodystrophy in an infant with homozygous E189X *BSCL2* mutation. Eur J Med Genet. 2009;52(1):14-16. 19041432 10.1016/j.ejmg.2008.10.006

[41] Janhavi S , Poovazagi V, Mohan V, et al Clinical and molecular characterization of neonatal diabetes and monogenic syndromic diabetes in Asian Indian children. Clin Genet. 2013;83(5):439‐445. 22831748 10.1111/j.1399-0004.2012.01939.x

[42] Abali ZY , De Franco E, Karalic Ozturan E, et al Clinical characteristics, molecular features, and long-term follow-up of 15 patients with neonatal diabetes: a single-centre experience. Horm Res Paediatr. 2020;93(7-8):423‐432. 33498041 10.1159/000512247 PMC7611806

